# Endometriosis-Related Ovarian Cancer: Where Are We Now? A Narrative Review towards a Pragmatic Approach

**DOI:** 10.3390/jcm13071933

**Published:** 2024-03-27

**Authors:** Gabriele Centini, Giorgia Schettini, Emilio Pieri, Matteo Giorgi, Lucia Lazzeri, Francesco Giuseppe Martire, Virginia Mancini, Diego Raimondo, Renato Seracchioli, Nassir Habib, Francesco Fedele, Errico Zupi

**Affiliations:** 1 Department of Molecular and Developmental Medicine, Obstetrics and Gynecological Clinic, University of Siena, 53100 Siena, Italy; centini.gabriele@gmail.com (G.C.); giorgiaschettini@gmail.com (G.S.); ep.emiliopieri@gmail.com (E.P.); lucialazzeri79@gmail.com (L.L.); francescogmartire@libero.it (F.G.M.);; 2 Department of Surgical Sciences, Gynecological Unit, Valdarno Hospital, 52025 Montevarchi, Italy; 3 Department of Surgical Sciences, Gynecological Unit, University of Rome “Tor Vergata”, 00133 Rome, Italy; 4 Department of Medical Biotechnology, Section of Pathology, University of Siena, 53100 Siena, Italy; virginia.mancini@unisi.it; 5 Division of Gynecology and Human Reproduction Physiopathology, IRCCS Azienda Ospedaliero-Universitaria di Bologna, 40138 Bologna, Italy; die.raimondo@gmail.com (D.R.); renato.seracchioli@gmail.com (R.S.); 6 Department of Medical and Surgical Sciences (DIMEC), University of Bologna, 40138 Bologna, Italy; 7 Department of Obstetrics and Gynecology, Francois Quesnay Hospital, 78201 Mantes-la-Jolie, France; dr.nassirhabib@gmail.com; 8 Department of Obstetrics and Gynecology, Fondazione “Policlinico-Mangiagalli-Regina Elena” University of Milan, 20122 Milan, Italy; francesco.fedele@unimi.it

**Keywords:** *ARID1A* mutations, atypia, atypical endometriosis, biomarkers, clear cell ovarian carcinoma, endometrioid ovarian carcinoma, endometrioma, *PI3K/AKT/mTOR* pathway, ultrasound, treatment

## Abstract

Background: Endometriosis affects more than 10% of reproductive-aged women, causing pelvic pain and infertility. Despite the benign nature of endometriosis, ovarian endometriomas carry a higher risk of developing endometrioid carcinomas (EnOCs) and clear cell ovarian carcinomas (CCCs). Atypical endometriosis, defined as cytological atypia resembling intraepithelial cancer, is considered the precursor of endometriosis-associated ovarian cancer (EAOC). This narrative review aims to provide an overview of EAOC, proposing a practical approach to clinical and therapeutic decision making. Methods: An electronic literature search was conducted from inception up to January 2023, using the MEDLINE database via PubMed to evaluate the existing literature on EAOC, including its pathogenesis, the diagnostic process, and the therapeutic possibilities, with articles not relevant to the topic or lacking scientific merit being excluded. Results: Eighty-one articles were included in the review to present the current state of the art regarding EAOC. A pragmatic clinical flowchart is proposed to guide therapeutic decisions and improve patient outcomes. Conclusions: Endometriosis patients may have an increased risk of developing EAOC (either EnOC or CCC). Despite not being fully accepted, the concept of AE may reshape the endometriosis–ovarian cancer relationship. Further research is needed to understand the unaddressed issues.

## 1. Introduction

Endometriosis is a chronic benign estrogen-dependent disease. It is characterized by the presence of endometrial glands and stroma outside of the uterus. It is a common public health problem [1], affecting up to 10% of reproductive-aged women and 30% of infertile patients [2,3,4,5]. Pelvic endometriotic implants tends to spread towards surrounding tissues, leading to fibrosis and tissue adherence. In some cases, endometriosis may metastasize to lymph nodes or beyond the abdominal cavity. The commonest site of disease is the ovary, often affected by a distinct ovarian cyst known as “endometrioma”. 

Although endometriosis is widely deemed as a benign disease, affected patients inherently have an increased risk of developing malignancy, especially these presenting with endometriomas [6]. Back in 1925, Sampson proposed an association between endometriosis and ovarian cancer, describing the development of an ovarian endometrioid carcinoma (EnOC) from ectopically implanted endometrial tissue [7]. This finding was later confirmed by Scott, who focused on the malignant changes in endometriotic tissues, pointing out that benign endometriosis (BE) might be contiguous with endometriosis-associated ovarian cancer (EAOC) [8,9]. 

The available evidence points to a direct relationship between specific subtypes of epithelial ovarian cancer and endometriosis. EAOC refers to a type of ovarian cancer that is believed to arise from or be influenced by endometriosis [9]. The term highlights the observed association between endometriosis and certain ovarian cancer subtypes, suggesting that endometriosis may predispose individuals to the development of these cancers or share common underlying factors.

This association has been validated through molecular pathology, demonstrating common mutations in cancer-associated genes. Although atypical endometriosis may precede these cancers, it is not consistently present in all cases of endometriosis-related ovarian cancer. 

Approximately 0.5–1% of cases of endometriosis are affected by different types of ovarian neoplasia, and EAOC occurs in 0.14–2.9% of individuals with endometriomas [10,11,12,13,14]. Among EAOCs, the most common histotypes are clear cell ovarian carcinoma (CCC), EnOC, and low-grade serous ovarian carcinoma [15]. Several studies have indicated atypical endometriosis (AE)—i.e., the histological finding of cytologic atypia and architectural atypia or hyperplasia—as the direct precursor to these specific tumor histotypes: AE is present in 12–35% of ovarian endometriosis cases, and approximately 60–80% of EAOC occurs with AE [9,16]. The mechanisms underlying the malignant transformation from BE to cancer are currently not well established, although various alternatives have been suggested, including excessive oxidative stress, altered cytokine production, genetic mutation occurrence, and the presence of a hyperestrogenic environment. These molecular mechanisms might become useful diagnostic targets for the early detection of endometriosis-related cancers. However, the clinical application of these novel biomarkers may pose challenges as they all require molecular analysis [9].

Therefore, considering the limited but existing risk of association and/or neoplastic degeneration of endometriomas, it is of fundamental importance to establish a diagnostic-therapeutic pathway aimed at investigating and identifying the presence of potentially malignant endometriotic ovarian lesions. Ultrasound imaging may be considered the first diagnostic tool that is useful in differentiating typical endometriosis from AE and EAOC, even though the only definitive diagnosis is the histological one. A correct diagnostic classification to identify “high-risk” disease would allow for the most appropriate treatment. A conservative pharmacological approach can usually be adopted to treat endometriotic lesions; however, surgery may be the first-line treatment option in cases where the risk of neoplastic degeneration is deemed concrete. Preoperative suspicion of a malignant transformation may lead to increased intraoperative care and efforts toward the prevention of intrabdominal cyst cell dissemination. 

The aim of this narrative review is to present the current state of the art of EAOC, offering a general view of the available data. The pathogenetic mechanisms of EAOC are reported, including the supposed precursory role of AE, as well as its challenging diagnostic and therapeutic pathways. We also propose a pragmatical clinical flowchart to optimize the available therapeutic options, favoring patient quality of life.

## 2. Materials and Methods

An electronic literature search was conducted to evaluate the existing literature on EAOC, encompassing the hypotheses for pathogenesis, the diagnostic assessment, and the potential therapeutic approaches. The search was performed using the online medical MEDLINE database (accessed via PubMed). A set of predefined search terms was employed, including the following: “Adenocarcinoma, Clear Cell” (MeSH Unique ID: D018262); “Atypia”; “Atypical”; “Atypical endometriosis”, “Biomarkers, Tumor” (MeSH Unique ID: D014408); “Carcinoma, Endometrioid” (MeSH Unique ID: D018269); “Diagnosis”; “Diagnostic Imaging” (MeSH Unique ID: D003952); “Endometrioma”; “Endometriosis” (MeSH Unique ID: D004715); “Genital Neoplasms, Female” (MeSH Unique ID: D005833); “Gynecologic Surgical Procedures” (MeSH Unique ID: D013509); “Pathogenesis”; “Therapy” (MESH unique ID: D013812); “Treatment”; “Ultrasonography” (MeSH Unique ID: D014463).

The investigation included articles from inception to January 2023. Original articles, including randomized and non-randomized clinical trials, prospective observational studies, retrospective cohort studies, and case–control studies, review articles, and case reports, were considered eligible for the purpose of this review. The research selection process began with a careful examination of articles’ titles and abstracts, ensuring that their content included elements relevant to our research question. Furthermore, we conducted a thorough examination of the bibliography of the selected articles, identifying additional papers for further scrutiny. The identified articles underwent a rigorous screening process conducted by three independent reviewers (G.C., L.L., and E.P.), who meticulously evaluated the content for relevance and scientific merit. Articles that deviated from the predetermined theme or lacked substantial scientific contributions were excluded. To ensure a more contemporary perspective on the subject matter, more dated articles were included only if historically relevant.

This methodological approach was used to compile a cohesive and high-quality collection of studies, providing a nuanced understanding of the specified topics within the context of EAOC research. 

Eighty-nine articles were included for the purpose of our narrative review, i.e., to present the current state of the art regarding EAOC.

## 3. Epidemiology and Pathogenesis of Endometriosis and Endometriosis-Associated Ovarian Cancers: How Can Endometriosis Progress into Cancer?

Different theories have been proposed to explain the pathogenesis of endometriosis, including retrograde menstruation, coelomic metaplasia, lymphatic or vascular dissemination, immune system dysfunction, genetic predisposition, and environmental impacts. These theories are not mutually exclusive, and it is likely that a combination of factors contributes to the development of endometriosis. However, a sequence of bleeding, inflammation, fibrin deposition, adhesion formation, and scarring and distortion of the peritoneal surfaces of organs and pelvic anatomy constitute the natural history of this disease [17]. 

Endometriosis is deemed a benign disease but has some features in common with the malignant ones. Indeed, it may have a metastatic behavior with attachment to the surrounding tissues, and in some cases, it metastasizes to distant organs. However, endometrioma represents the most common presentation of the disease and is considered a benign ovarian cyst [5,6]. Nevertheless, the presence of endometrioma determines an increased risk, albeit an overall limited risk, of concomitant ovarian malignancy in the affected patient [9], with these malignancies often arising from the endometriotic tissue itself. Indeed, two different scenarios have been proposed to explain the malignant progression of endometrioma into EAOC.

The first one is a cyclic hemorrhage occurring inside the endometriotic cyst that leads to the accumulation of blood components (i.e., extracellular hemoglobin, iron, and heme), inducing cellular oxidative damage by elevating reactive oxygen species. This oxidative stress induces DNA damage and subsequent oncological mutations. 

The second scenario revolves around the continuous production of antioxidants: endometriotic cells adapt to oxidative stress with the aid of macrophages, enhancing antioxidative defenses and influencing redox signaling, energy metabolism, and the tumor immune microenvironment, potentially leading to malignant transformation [18]. Additionally, some specific molecular alterations have also been noted, such as *ARIDA1/BAF250a*, *PIK3CA*, *CTNNB1*, and *PTEN* mutation, as well as microsatellite instability and the loss of heterozygosity [19,20,21,22,23].

Given that endometriosis is typically not associated with cancer, we can hypothesize that the mechanisms of cellular oxidative damage are self-restricting in the majority of patients. This phenomenon maintains a pro-inflammatory environment characterized by a delicate equilibrium between oxidative stressors and antioxidant mediators [18,19,20,21,22,23,24]. Nevertheless, there is no established molecular mechanism that can be used to predict, with certainty, the oncological progression of the disease in these patients, making it challenging to identify at-risk patients early on. The role of cancer-driving mutations and the correlation between genotypes and clinical outcomes are still to be elucidated [25].

However, some clinical risk factors for the development of EAOC among patients with endometriosis have been identified: older age at the time of diagnosis, presence of a solid component inside the cyst, postmenopausal status, large-sized (≥9 cm) endometrioma, nulliparity, and hyperestrogenism [26]. 

### Epidemiological and Prognostic Characteristics of the Main Endometriosis-Associated Ovarian Cancers Histotypes 

**Figure 1:**
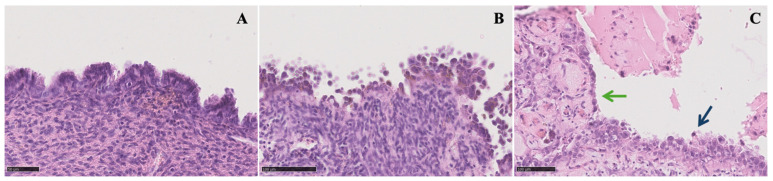
Microscopic view of different grades of endometrioma atypia (50–100 μm): (**A**) typical endometrioma is lined by endometrioid epithelium with no endometrial stroma beneath it; (**B**) endometrioma with atypia showing nuclear pleomorphism, an inverted nucleus-to-cytoplasmatic ratio, and abundant eosinophilic cytoplasm with epithelial stratification and tufting; (**C**) endometrioma with foci of atypia (green arrow) and foci of clear cell carcinoma (blue arrow).

EAOC typically impacts women in the age range of 35–55 years. Approximately 0.5–1% of cases of endometriosis are complicated by neoplasia, and EAOC is observed in 0.14–2.9% of individuals with endometriomas [8,9,10,11,12]. Among EAOCs, the most prevalent histotype is CCC, which accounts for 5–12% of cases; it exhibits geographical variability, being more common in certain Asian countries. It is characterized as a high-grade ovarian carcinoma and is associated with a poor prognosis in advanced stages due to its early resistance to platinum-based treatments. The second most common histotype is represented by EnOC, which constitutes about 10% of EAOCs; the third most frequent EAOC is low-grade serous ovarian carcinoma [15,27]. The majority of CCCs and EnOCs fall under the category of Type I ovarian tumors, originating from benign lesions implanted on the ovary and undergoing subsequent malignant transformation (within benign ovarian endometriotic cysts in the case of endometriosis). Type I ovarian tumors are typically clinically indolent and characterized as low-grade carcinomas [21,22] (Figure 1).

## 4. Atypical Endometriosis

AE is present in 12–35% of ovarian endometriosis [16], and approximately 60–80% of EAOC occurs with the concomitant presence of AE (in 23% of EnOCs and in 36% of CCCs) [28], often in direct continuity with the tumor [29]. The high variability of the incidence might be attributed to its difficult histological diagnosis, which lacks worldwide uniformity. Therefore, it is clear that there is a need to revise the classification in order to identify histologically “high-risk” diseases. 

Several studies have considered AE as a direct precursor to CCC and EnOC. This hypothesis is based on specific histologic criteria detected on AE specimens, including large nuclei, significant pleomorphism, an increased nucleus-to-cytoplasmic ratio, cellular crowding, and stratification [18,19,28,29].

We may consider the hypothesis that premalignant lesions like AE may act as an intermediary stage along the pathway to cancer, reflecting genetic changes that occur prior to the onset of malignant behavior. The presence of frequent mutations in cancer-associated genes confirms this association at the molecular pathologic level. In EnOC, mutations in *CTNNB1*, *PTEN*, and *ARID1A* are common, while in CCC, *PIK3CA* and *ARID1A* mutations are prevalent [30]. Even if AE may be the precursor of these cancers, it is not systematically found in all cases of EAOC [31].

Three distinct scenarios may arise: ovarian cancers with histological evidence confirming the transition from endometriosis to ovarian cancer, as defined by Sampson and Scott; the coexistence of ovarian cancers with endometriosis in the same ovary, lacking histological proof of transition; and the occurrence of ovarian cancers alongside concurrent endometriosis at any pelvic location [16].

AE includes two distinct histologic findings: cytologic atypia and architectural atypia or hyperplasia [20]. The term “cytologic atypia” refers to the presence of abnormal nuclear features in the epithelial lining of endometriotic cysts, whereas “architectural atypia or hyperplasia” represents the same range of abnormal cell growth found in the endometrium [30]. The identification of architectural atypia in endometriosis is important because patients with hyperplastic AE may be at an increased risk of developing EAOC [31].

Tanase et al. highlighted the need to recognize the potential malignant transformation, strongly advising the diligent monitoring of AE when it is detected. They reported the case of a 33-year-old woman whose condition evolved over the span of 10 years and three laparoscopic surgeries from typical endometriosis to AE and eventually to EnOC [32].

Nevertheless, there is a scarcity of evidence about clinical characteristics, risk factors, and the likelihood of AE recurring. Hence, given that AE is characterized by histological premalignant changes, it is acknowledged as having the potential for precancerous progression, but some mechanisms driving malignant transformation remain uncertain, though several pathways from BE to cancer have been proposed. These pathways implicate factors such as oxidative stress, cytokine activity, genetic mutations, and exposure to a hyperestrogenic environment.

Furthermore, it should be noted that while some studies explain AE as reactivity to severe local inflammation or superficial ulceration with regenerative activity, possibly leading to dysplasia, the association with severe stromal inflammation remains contentious. Conversely, other authors indicate that epithelial atypia can manifest independently of inflammation, implying an intrinsic precancerous potential [33]. 

Notably, AE and EAOC share common molecular/genetic alterations, including somatic *ARID1A* [32,34], *PTEN* [35], and *PIK3CA* mutations [36]; *HNF-1b* up-regulation [37]; the loss of the estrogen and progesterone receptor [38]; and, rarely, *TP53* mutations [39]. These mutations delineate a spectrum of tumor progression, evolving from benign cystic neoplasms to their corresponding carcinomas. This progression is frequently evident through precursor lesions such as AE (Table 1). It is worth noting that while atypical endometriosis poses an elevated risk of malignant progression, instances of transformation into carcinoma are infrequent and warrant further investigation.

Several potential targets, such as these molecular changes, have been suggested for the early identification of cancers linked to endometriosis. Yet, the practical use of these new biomarkers could present hurdles, as they all necessitate molecular analysis.

## 5. EAOC and Endometrial Cancer

Fifty percent of individuals with EnOC show concomitant endometrial adenocarcinoma, making detailed ultrasonographic endometrial assessment imperative when EnOC diagnosis is suspected. In 2–8% of patients affected by endometrial adenocarcinomas, there is the potential for synchronous ovarian carcinoma, necessitating thorough ovarian evaluation in conservative endometrial adenocarcinoma treatments. Notably, approximately 90% of concomitant tumors involving both the ovary and the endometrium display endometrioid histology. Research indicates a substantial prevalence of coexisting endometriosis in cases presenting with simultaneous ovarian and endometrial carcinoma. Patients with EAOC display a higher rate of synchronous endometrial cancer, reporting a reduced recurrence rate and enhanced 5-year disease-free survival, although this phenomenon does not translate into a discernible difference in overall survival [42].

## 6. Clinical Approach: How to Make a Diagnosis and Issues for Early Cancer Detection

In patients with endometriotic cysts, preoperative suspicion of malignant transformation is essential to prevent the intraoperative dissemination of malignant cells. To achieve this goal, a considerable level of proficiency in transvaginal sonography (TVS) is necessary, as it remains the most advantageous and easily accessible approach for preoperative evaluation, being a real time dynamic assessment with a sensitivity ranging from 79 to 94% and a specificity of 94% [43]. MRI may provide supplementary information in specific instances, but it is not a routine method for the preoperative evaluation of endometrioma. The tumor marker CA-125 may be of assistance, although its diagnostic value for early-stage EAOC is restricted due to its lack of specificity. Notably, it is worth mentioning that women with BE often experience slightly increased levels of CA-125, even in the absence of any signs of EAOC [25,27,44,45,46].

Sonography continues to be the major technique for evaluating the risk of cancer. Distinguishing between endometriomas and early-stage cystic CCC or EnOC through sonography can be quite challenging. In some cases, a carcinoma may develop from an abnormal epithelial spot within an endometrioma, being difficult to recognize by sonography in its early stage [47,48,49,50,51].

Even though endometriosis is increasingly prevalent among young women, EAOC primarily occurs in elderly women [52] and is characterized by the presence of solid components and larger tumor sizes [10,26,44]. Nevertheless, as individuals grow older, the occurrence of endometriomas containing blood clots miming solid components becomes more common [25,44,53]. During follow-up, these lesions may develop additional atypical features, such as a larger size or multi-cystic formations [25,53]. The Ovarian Tumor Analysis (IOTA) database has shown that approximately 21% of endometriomas in women who are 45 years or older may contain solid components [53]. Furthermore, although ovarian CCC is commonly detected in its early stages, conventional imaging indicators for malignancy may have restricted diagnostic significance [54,55]. The lack of guidance on differentiating endometriomas from CCCs is a critical matter that requires immediate attention [27].

### 6.1. Ultrasound

**Figure 2:**
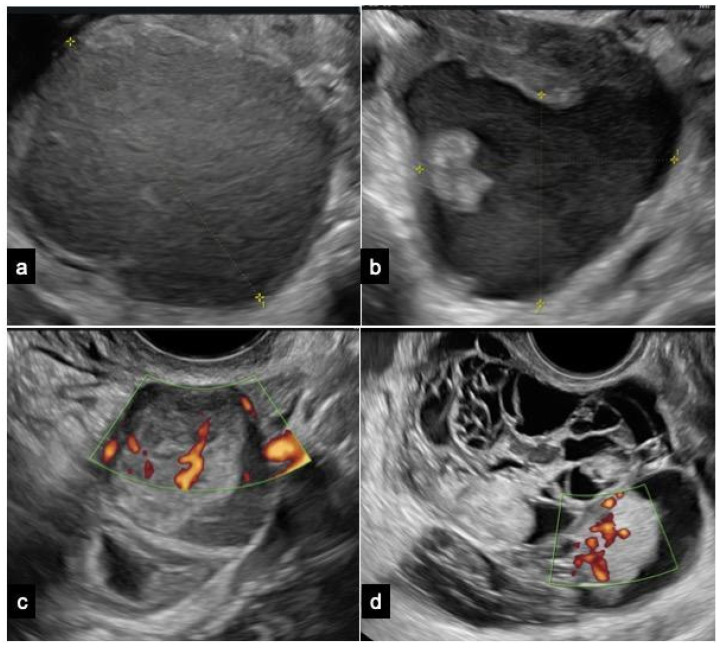
Ultrasound images of (**a**) a typical endometrioma, (**b**) an atypical endometrioma, (**c**) a clear cell carcinoma, and (**d**) an endometrioid carcinoma. The ultrasonographic diagnosis was confirmed by pathological examination, and patients provided informed consent for the use of their images.

Endometrioma is predominantly a unilocular cyst displaying a uniform “ground-glass” echogenicity without observable solid or vascularized papillary components, facilitating its diagnosing in non-experienced hands (Figure 2).

However, endometrioma can be defined as atypical when at least one of the following sonographic characteristics is observed: cyst diameters of 10 ± 1 cm, multi-cystic formations, the presence of any solid component or papillary structure—defined by IOTA as a protrusion of solid tissue into a cyst cavity with a minimum height of 3 mm—and the detection of blood flow at any level [56,57]. The IOTA risk score considers unilocular cysts with small solid components (less than 7 mm of maximum diameters), acoustic shadows, uniform multilocular tumors with maximum diameters less than 10 cm, and the absence of blood flow as benign features. Malignant features encompass irregular solid tumors, ascites, four or more papillary structures, irregular multilocular solid tumors with a maximum diameter ≥10 cm, and strong blood flow. Typically, obtaining a histological evaluation post intervention for atypical endometriomas deemed at neoplastic risk is recommended. However, additional considerations, including patient’s age or fertility desires, are essential for determining the most appropriate management approach [56,58]. 

Ovarian CCC is typically diagnosed in its early stages, presenting as a sizable unilateral mass with solid components. Patients with CCC arising from endometriosis tend to be younger compared to those with non-endometriosis-associated clear cell carcinoma. Moreover, CCC originating from endometriosis may frequently display a ground-glass echogenicity in the cyst fluid [59].

EnOCs typically present as larger, unilateral, multilocular-solid, or solid tumors. The ultrasound features of EnOC originating from endometriosis exhibit distinctions from those not associated with endometriosis [52].

Borderline tumors and carcinomas originating from endometriomas typically show a vascularized solid component. An age of 45 years or older and endometrioma size of 8 cm or more are significant factors that independently predict the development of ovarian cancer in women with endometriomas [10,22,31]. 

A promising approach for the early diagnosis of endometrial and ovarian cancer seems to be represented by investigating mutation analyses in endocervical or, preferably, intrauterine cell samples. Nevertheless, additional research is needed to determine the accuracy and reliability of these approaches in a clinical setting. Even if, in the future, certain methods may be successful for a peri/postmenopausal population, they may not necessarily be applicable to younger patients with endometriosis, due to the frequency of occurrence of genetic mutations in both eutopic and ectopic endometrium being greater previously assumed [31,60,61,62,63]. Currently, there are no effective screening options for epithelial ovarian cancer, and this holds true for women with endometriosis as well [64,65,66,67]. 

Early-stage disease with subtle morphological changes poses a challenge for the current diagnostic modalities, and histological examination, though definitive, is invasive. 

### 6.2. Other Instrumental Exams: MRI and CT

In the event of uncertainty following an expert sonographic assessment, additional instrumental examinations are available. Consideration may be given to performing magnetic resonance imaging (MRI) with contrast to differentiate benign ovarian formations from borderline and malignant ones. If neoplastic suspicion is confirmed, a computed tomography (CT) scan of the thorax–abdomen–pelvis at the earliest opportunity is imperative. The objective is to assess the intraperitoneal diffusion of the suspicious mass, lymphadenopathy, and ureteral stenosis in the retroperitoneal space, along with potential thoracic diffusion involving pleural, parenchymal, or mediastinal nodules.

CT is recommended for the staging of ovarian cancer. Contrast-enhanced CT provides clinically relevant information, including the size of the primary tumor and the size and location of any peritoneal and lymph node implants. This information is integral for predicting resectability. The overall accuracy of contrast-enhanced CT in diagnosing malignant neoplasms reaches 89%. CT can visualize tumor implants larger than 1 cm with a sensitivity ranging from 85 to 93% and a specificity of 91–96%. However, the sensitivity diminishes to 25–50% when detecting implants 1 cm or smaller [68].

MRI offers excellent tissue differentiation and serves as a valuable tool for characterizing lesions that may contain fat observed upon CT or ultrasound with indeterminate significance. Overall, MRI exhibits an accuracy of 83–91% in distinguishing between benign and malignant ovarian masses. The staging accuracy of MRI is comparable to that of conventional CT [43]. Due to its superior resolution in soft tissue contrast, MRI can precisely identify the invasion of pelvic organs. In predicting resectability, MRI demonstrates a sensitivity of 94% and specificity of 77%, in contrast to 55%, 86%, and 63%, respectively, for CT.

### 6.3. Molecular Biomarkers

Molecular biomarkers may also be used for further evaluation when the histopathological investigation is not sufficient for assessing the presence of EAOC. For instance, *Ki-67* specifically binds to a nuclear nonhistone protein that is present in the nuclei of actively dividing cells. It can forecast the likelihood of AE developing into a precancerous condition. Statistical differences were discovered by Ogawa and colleagues in the *Ki-67* indices of typical endometriosis, AE, and ovarian cancer [69]. Their findings indicate that AE shows a level of proliferation activity that falls between typical endometriosis and ovarian carcinoma, positioning it as a precancerous condition (with *Ki-67* indices of 2.7 ± 0.90, 9.9 ± 1.73, and 23.1 ± 3.29, respectively). 

In addition, *CD10* can assist in identifying endometriosis in cases where it is challenging to detect through histology, assessing endometriotic tissue characterized by the presence of endometriotic stromal cells [70].

BE often experiences slightly increased levels of CA-125, even in the absence of any signs of EAOC, but its value usually stays below 100 U/mL. Despite extensive study, CA-125’s diagnostic efficacy for endometriosis remains limited due to its low sensitivity (20–50%). Current international guidelines do not recommend routine CA-125 measurement in endometriosis diagnostics. Elevated CA-125 levels are associated with severe forms of endometriosis and the progression of endometriosis, particularly in ovarian endometriomas (stages three and four). Moreover, CA-125 levels tend to decrease following both medical and surgical interventions for endometriosis.

Patient education regarding the significance of an elevated tumor marker without malignancy is crucial, considering the emotional impact. The decision to categorize the condition as benign or potentially malignant requires careful discussion. Theories propose that CA-125-rich fluid within an endometriotic cyst, particularly after leakage, contributes to elevated levels. This fluid, upon absorption into the peripheral circulation, can induce peritoneal inflammation, leading to an elevated CA-125 level. Additionally, heightened peritoneal fluid in mild endometriosis, with CA-125 concentrations surpassing serum levels, may contribute to elevated serum CA-125 measurements [71,72,73]. 

In 2008, Yamaguchi et al. identified elevated iron levels in endometriotic cysts, proposing a potential diagnostic marker [74]. Recent advances include the non-invasive quantification of iron levels using MRI relaxometry. This technique, demonstrating high sensitivity and specificity, holds promise for the early detection of malignant transformation in endometriosis, offering valuable insights for disease management strategies [75].

## 7. Common Pathogenic Features of Endometriosis and EAOC, Novel Biomarkers, and Potential Target Therapy

Certainly, AE and EAOC share common molecular/genetic alterations, including somatic *ARID1A* and *PTEN* mutations, *PIK3CA* mutations, *HNF-1b* up-regulation, the loss of the estrogen receptor and progesterone receptor, and, occasionally, *TP53* mutations. These mutations illustrate the spectrum of tumor progression from benign cystic neoplasms to corresponding carcinomas like EnOC and CCC, often evolving through precursor lesions such as AE. While various targets have been proposed for the early detection of endometriosis-related cancers, implementing these novel biomarkers in clinical practice may be challenging due to the requirement for molecular analysis [19].

The occurrence of frequent mutations in *ARID1A*, associated with the *SWI/SNF* complex, has led to extensive research on their involvement in ovarian CCC, EnOC, and their precursor lesions [7,27,76]. *ARID1A* mutations, found in approximately 60% of CCCs and 30% of EnOCs, lead to a loss of function, primarily seen in CCC and BE cases. Significantly, endometriosis is the sole benign condition where a deficiency in *ARID1A* expression has been detected, even in situations where there is no indication of malignancy [29,52,77,78,79,80,81].

Although *ARID1A* inactivation occurs early in the process, it is not enough to cause cancerous transformation. Additional mechanisms, such as *PIK3CA*-activating mutations, are necessary for this purpose [27,82,83]. Sequencing-based detection is difficult, therefore making *ARID1A* immunohistochemistry a valuable substitute indicator. It is important to note that there are currently no specific genetic mutations that can be used to differentiate between CCC and EnOC, even though they have different physical and clinical characteristics. CCC exhibits genomic features in 26% of cases, while microsatellite instability is prevalent (28%) in EnOC [27,84]. 

Targeting *ARID1A* mutations directly for therapeutic purposes is not feasible [77], thus driving the investigation of the synthetic lethality strategy to target cancers with *ARID1A* deficiency [85,86]. *ARID1A* mutations frequently coincide with *PI3K/AKT* pathway activation in CCC, indicating a collaborative function in the process of malignant transformation. Preclinical studies demonstrate synthetic lethality using inhibitors like MK-2206, perifosine, buparlisib, AZD8055, and the *HDAC6* inhibitor ACY1215. Synthetic lethality in *ARID1A*-mutated tumors can be induced by targeting *ARID1B* [76,77]. Chronic inflammation, driven by *ARID1A* loss and *PIK3CA* mutations, contributes to CCC development through sustained IL-6 production. Anti-IL-6 therapies may be effective. Immune checkpoint inhibitors successful in *SWI/SNF*-related cancers show promise in CCC. The *SWI/SNF* complex is crucial for DNA damage repair and oxidative stress resistance, making *ARID1A*-mutant tumors more sensitive to reactive oxygen species-inducing agents, leading to apoptosis. Dasatinib, a multi-inhibitor, selectively targets *ARID1A*-mutated CCC, causing cell cycle arrest and apoptosis [78,79].

Potential therapies for *ARID1A*-deficient CCC involve targeting proliferative pathways (*PI3K/AKT/mTOR*, *YES1/SRC*) and metabolic alterations (glutathione biogenesis). Clinical trials explore agents demonstrating synthetic lethality. While *ARID1A* mutations show promise as predictive biomarkers, their role in early cancer detection and other biomarker studies is under investigation. Conflicting results in studies on *ARID1A* as a prognostic marker in ovarian cancer highlight the need for further research [27,80,81,82,83,84,85,86]. Clinical trials explore new therapeutic options for ARID1A-mutated tumors, including *ATR* and *PI3K/Akt/mTOR* pathway inhibitors. Everolimus plus bevacizumab shows potential benefits, particularly in ARID1A-mutated ovarian cancer. Dasatinib, already approved for leukemia, is currently being investigated for its potential use in treating different solid tumors. Ongoing trials are assessing novel *EZH2*, *HDAC*, and *BET* inhibitors. *ARID1A* status may guide chemotherapy decisions, with gemcitabine showing effectiveness in platinum-resistant CCC, especially in *ARID1A*-deficient cases.

Furthermore, Moga et al. investigated circulating miRNAs as potential non-invasive diagnostic biomarkers for endometriosis and EAOC, as they are key regulators of cellular processes. Despite their inherent limitations, miRNAs guarantee simplicity, tissular specificity, and steadiness in biological fluids [87]. *MiR-200* family (*miR-200a*, *miR-200b*, *miR-141*) dysregulation in endometriosis suggests a significant role in the disease’s pathophysiology. Noteworthy miRNAs, including *miR-20a* and *miR-143*, show potential implications for lesion growth and cellular invasion. *MiR-199a*, downregulated in endometriosis, has controversial diagnostic potential, likely *miR-145* regulation, with conflicting reports across studies and disease stages.

## 8. Surgery

A conservative pharmacological approach is usually preferred for the treatment of endometriotic lesions according to symptoms, age, patient desire, and contraindications [5]. However, it is essential to identify cases that warrant surgery even if asymptomatic, thereby minimizing the risk of neoplastic degeneration associated with certain types of lesions. Detecting the rare cases with an elevated risk of malignant transformation before surgery is crucial in patients with presumed endometriotic cysts in order to prevent the intraoperative dissemination of malignant cells [25].

The indication for the surgical management of AE is not straightforward and poses decisional challenges, with the need to evaluate several clinical factors to assess the risk of malignancy, including age, medical history, pregnancy desire, endometriotic cyst history, non-reassuring sonographic characteristics (papillary projections, septa, positive Color Doppler), and tumor biomarkers. 

In favor of the surgical approach, Melin et al. showed a significant reduction in future ovarian cancer risk, leading to the complete surgical removal of endometriosis lesions, including endometriomas [24]. As for any case of adnexal mass with the suspicion of cancer, when AE is coexistent or followed by suspected EAOC, a more aggressive approach might appear to be the safest route. However, the line between under- and over-treatment is very thin, with there being a high risk of crossing it. 

Due to the relatively low incidence of neoplastic association and/or the transformation of endometriomas, unilateral salpingo-oophorectomy (USO) cannot be universally recommended for all cases. Such a practice may reflect an excessive level of caution and could easily lead to over-treatment. This is even more true considering that premenopausal oophorectomy is associated with increased all-cause mortality and significant menopause-related morbidity, decreasing life expectancy [88]. Furthermore, based on Melin’s analysis, performing systematic USO on women with endometriomas would prevent only one case of ovarian cancer every 62 interventions [24]. It is worth mentioning that their estimates referred to the removal of every endometriotic cyst—including benign endometriomas—not just the atypical ones that are the basis of our assessments.

The risks and benefits of USO should be thoroughly discussed with the patient affected by AE and evaluated independently from her age. Although not recommended in general due to the associated health consequences, USO may be a viable option in adequately informed women approaching menopause with no desire of pregnancy. It may be offered especially when EAOC risk is higher due to patient history (positive family history of ovarian cancer or previous history of infertility), cyst history (premenopausal newly diagnosed atypical endometrioma, endometriotic cyst size increase, long-term cyst with no previous hormonal contraceptive treatment), and/or suspicious imaging characteristics. According to Vercellini et al., 45 years of age could be considered a valuable age cut-off for estimating one’s premenopausal status irrespective of symptoms suggestive of climaterium [25]. 

**Figure 3:**
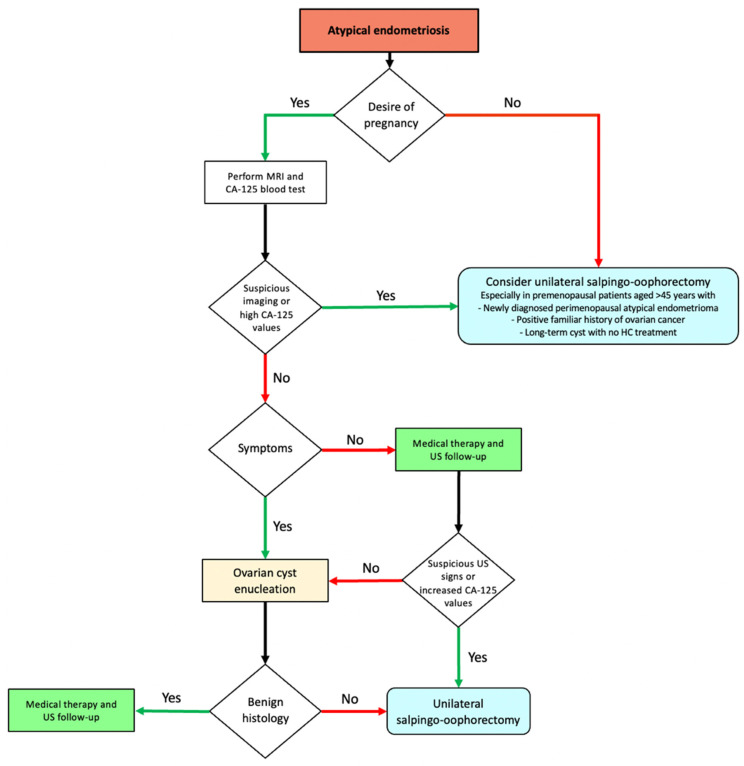
Flowchart for surgical management of ovarian atypical endometriosis. Abbreviations: MRI, magnetic resonance imaging; HC, hormonal contraceptive; US, ultrasound.

Conversely, USO should not be considered the first choice for young women desiring pregnancy. Since CA-125 is already increased in BE, it may not effectively distinguish atypical endometriomas from EAOC; in these cases, monitoring ultrasound characteristics is crucial for optimal decision making. MRI may offer additional value in specific cases: in instances where atypical sonographic features are detected, the exclusion of malignancy-associated elements may be achieved through MRI. A fertility-sparing approach may include either a conservative cyst excision or a wait-and-see follow-up, provided that the clinical characteristics of the cyst permit it (for example, if the cyst does not increase in size over time and the atypical ultrasound findings remain stable). The optimal follow-up timing is still unknown for atypical endometriomas, probably due to the scarcity of data. Further information is needed to shed light on the unclear aspects regarding EAOC (Figure 3).

Keeping the patient informed through up-to-date and comprehensive counseling is essential for effective management. Physicians must carefully weigh the risks of over- and under-treatment, providing patients with the necessary information to make informed decisions about their care.

Surgery must always be followed by careful follow-up. Indeed, according to some studies, women experiencing a recurrence of endometriotic lesions after excision have an elevated risk of ovarian neoplasia. This particular patient population must be offered close personalized follow-up and a possible second operation [89].

Finally, in consideration of the most recent data, it would be interesting and desirable in the future to investigate possible therapeutic alternatives that could be effective in reducing recurrence after surgery in order to further reduce the neoplastic risk.

## 9. Conclusions

Our narrative review summarizes the current knowledge on EAOC, proposing a flowchart dedicated to the management of the disease. Endometriosis patients appear to have an increased risk of developing EAOC, particularly the EnOC and CCC subtypes. However, the scarcity of data currently prevents definitive conclusions.

Researchers still need to understand some unclear aspects, such as early detection, risk factor identification, and risk stratification. Advances in the *SWI/SNF* complex and *ARID1A* alterations provide insights into endometriosis and EAOC carcinogenesis. The success of *PARP* inhibitors in ovarian cancer therapy fuels the exploration of new targeted therapies. Inflammatory and epigenetic processes, prominent in the CCC and EnOC subtypes of EAOC, suggest potential treatments with immune checkpoint inhibitors, *PI3K* pathway targeting, and epigenetic approaches. Clinical research tailored to the molecular features of these subtypes will be crucial.

Finally, it is worth underlining that TVS diagnosis remains the cornerstone of early detection, guiding the patient towards the most appropriate therapy, follow-up, and treatment, while MRI aids in characterizing TVS-indeterminate lesions. Malignancy indicators, such as increasing tumor size, rapid growth, papillary excrescences, and thick septations, are crucial for suspicion.

## Figures and Tables

**Table 1:** Prevalence of epithelial ovarian cancer histotypes and associated genetic mutations [40,41]. Abbreviations: OC, ovarian cancer.

OC Histotype	Category	Proportion of OC	OC Genomics
High-grade serous	Type II	75%	*TP53* (≥95%) *BRCA1* or *BRCA2* (~20%) *CCNE1* (14%) *EMSY* (~15%) *non-BRCA HRR* (<5%) *NF1* (<5%) *PTEN* (<5%) *RB1* (<5%)
Endometrioid	Type I	10%	*CTNNB1* (30–50%) *PIK3CA* (30–50%) *PTEN* (30–45%) *KRAS* (25–40%) *ARID1A* (20–40%) *TP53* (11–24%) *MMR* (8–19%)
Clear cell	Type I	10%	*ARID1A* (40–50%) *PIK3CA* (40–50%) *PPP2R1A* (10–20%) *SYNE1* (~20%) *KRAS* (5–20%) *TERT* (5–15%)
Low-grade serous	Type I	≤5%	*KRAS* (15–55%) *BRAF* (0–33%) *USP9X* (13–27%) *NRAS* (4–22%) *EUF1AX* (6–15%) *CDKN2A* *ERBB2* *PIK3CA*
Mucinous	Type I	≤5%	*TP53* (60–70%) *KRAS* (60–70%) *CDKN2A* (50%) *ERBB2* (25%) *BRAF* (~10%) *PIK3CA* (~10%)

## Data Availability

Not applicable.
